# A Large Prize Offered

**Published:** 1854-07

**Authors:** 


A Large Prize Offered.—The United States Department of State has published a letter from that indefatigable Frenchman, Mr. Vattemare, addressed to John Y. Mason, which the latter gentleman transmitted to Secretary Marcy, accompanied with a letter from himself. Mr. Vattemare, by his will, leaves $100,000 to any person who discovers the "means of curing Asiatic Cholera, or of the cause of the pestilence." To give publicity to the fact, the publication has been made. The power of awarding the prize has been conferred on the Institute of France, and the interest of it, until it has been awarded, is to constitute an annual prize, to be given to those who advance the knowledge of the cause of Cholera and its remedy.

